# The Week's Work

**Published:** 1910-06-18

**Authors:** 


### The Week's Work.

### THE ATTACK ON ST. BARTHOLOMEW'S HOSPITAL

The Nursing Mirror this week contains a facsimile copy of the certificate of Miss McIntosh, whose appointment as matron has given rise to discussion. That certificate and inquiries at the London Hospital prove that Miss McIntosh has had three years' practical training in the London Hospital. The critics of the appointment use the term "practical service in the wards and the necessary theoretical instruction." No modern and up-to-date training school for nurses is efficient which does not (1) provide strict supervision over the progress of all probationers during the period of training, and (2) differentiate between those who are capable of qualifying for the higher nursing posts and those who are not. However much may be said in favour of three years devoted entirely to work in a hospital ward for the latter class of probationers, no practical authority whose opinion is worth having will contend that the higher type of probationer can in fact have an adequate training during her three years' course unless some of it is devoted to giving her an opportunity to learn the duties of every branch of hospital work, including those of the matron herself. It is for this reason that the training of Miss McIntosh at the London Hospital during the three years to which her certificate extends is one which does credit to the London Hospital. It proves that in her case the system we have indicated is wisely pursued in the training of probationers who aspire to fit themselves for the higher positions in the nursing world.

### ROYAL ALBERT EDWARD INFIRMARY, WIGAN.

In calling attention to Sir Henry Burdett's report on this institution, published in our issue of May 28, the *Wigan Examiner* properly emphasises the fact that this institution is defective for the reason that the present arrangements for the medical, nursing, and domestic staffs are unworthy of, and unfair to, the devoted workers who have made the Wigan Infirmary the comfortable and efficient hospital it is to-day in other respects. The *Wigan Examiner* emphasises the devotion of the staff, and we desire to-day to try and persuade the authorities to put in hand at once the following works, which they owe it to the reputation of their hospital to proceed with without delay: (1) At the present time there is only one general sitting-room for the whole staff, 41 in number; (2) the assistant matron has no sitting-room or office of any kind to do her work in; (3) there is no recreation-room, dining-room, or sitting-room of any kind provided for the domestic staff of 27 servants, and they have only one bath and one indoor lavatory set apart for their use. These things should not be, and the fact that they exist in the Wigan Infirmary is the more remarkable because the condition of the wards testifies to the fact that all the members of the staff are loyal and most efficient. We desire to impress upon the committee that, however urgent it may regard the necessity for a new out-patient department, no expenditure upon such a building is justifiable until the nursing and domestic staffs have been properly and comfortably housed. It is due to the high reputation of the hospital and the committee itself that not a day should be lost before this business is taken in hand and properly accomplished. Will the committee and the people of Wigan please remember that an up-to-date modern hospital provides each nurse with a bedroom to herself, and that the servants' quarters do not consist of cubicles, much less of small cubicles barely large enough for one person, which contain two beds? We are quite sure, in view of the reception accorded to Sir Henry Burdett's report, that we have only to call the attention of the committee and the supporters of the Wigan Infirmary to the imperative necessity that the defects here pointed out shall be removed from the Wigan Infirmary to ensure that they will be faced and the whole of these matters put right at once. No doubt the local Press, which has an opportunity of examining the condition of the quarters referred to, will lend a hand on behalf of the members of the devoted staff, who ought not longer to be allowed to suffer, as they must be suffering, from the defects in question.

### THE EAST SUFFOLK AND IPSWICH HOSPITAL.

The East Suffolk and Ipswich Hospital has published an interesting little guide to the institution. This little handbook, which is a model of what such things should be, gives a description of the hospital as it appears to-day, with full plans and, what is specially worthy as a novel feature, a map showing the district which the institution serves. The letterpress is concise and clear, the descriptions being simple and easily understood by the lay reader who has no great knowledge of technical terms. The photographs with which the guide is illustrated are especially good, the reproductions in every case being excellent, while the subjects are well chosen. This little book is no report; it is merely a handy reference guide such as Sunday and other visitors to the hospital may use when they inspect the institution. There is no lengthy list of subscribers; no long introduction, historical or otherwise. While the East Suffolk Hospital is not the first to adopt the plan of issuing such a guide, it is to be congratulated upon being the first institution thoroughly to grasp the fact that attractiveness and real utility are the primary considerations which should weigh with the publishers if such a pamphlet is to be of real value to visitors and friends of the charity.

### HOSPITAL SUNDAY.

Hospital Sunday this year passed quietly, and was marked by no special features in spite of the earnest appeal which was made on behalf of the Fund. A walk round the metropolitan district, had anyone been energetic enough to undertake it on a day so excellent for outdoor recreation as Hospital Sunday this year proved to be, would have shown the majority of churches hardly so full as one would have expected in view of that appeal. Whether the congregations were more generous, and, as a result, whether the sum total of the contributions exceeds that of former years, remain to be seen. We can only hope that the public has responded nobly to the call, and we are strengthened in that hope by the knowledge that in some churches, at least, the gifts in aid of the Fund have been most generous and cordial. Thirty-seven years ago the first collections on behalf of Hospital Sunday were made, and since that time the good work that the Metropolitan Sunday Fund has been able to do has received general recognition. What the public ought to understand, however, is that there is a real fear that this good work, done all these years so impartially, steadily, and successfully, will have to be curtailed in some respects unless a more generous support is extended to the Fund. There has been a steady falling-off in the total amounts collected during the last five years, and this, as we have previously pointed out, notwithstanding the fact that the number of contributing churches has increased.

### A SUGGESTION.

It is perhaps worth while to reconsider the arrangement which has so far been followed, under which the Sunday collection for the Fund is held in summer. Summer attendances at church are on the average lower than during the winter months, and they are certainly very much lower in London. When it is borne in mind that a large number of metropolitan churchgoers live outside the metropolitan area during the months of June, July, and August, and that a far larger proportion attend at seaside and country places of worship during these months, owing to the fact that "week-end" visits are annually becoming more popular, the gradual diminution in the collections taken in the metropolitan area on Hospital Sunday is scarcely to be wondered at. The question is how can this state of affairs be dealt with so as to improve the value of the collections made within the metropolitan area on Hospital Sunday? Ordinary appeals are useless: it is too much to ask the metropolitan worker to give up his week-end visit for the sake of the Fund, though there are some who might reasonably be appealed to for substantial support of the Fund even if they are not able actually to be present when the church collections are taken. At present the main point is that many metropolitan churchgoers worship in country churches on Hospital Sunday, and that they are thus unable to contribute directly to the collections, while owing to the absence of any reference to the work of the Fund on such days by the country clergy, they are for the most part ignorant of the fact that it is Hospital Sunday, and that they have certain duties and obligations as citizens towards the Fund. It would be well if the country clergy made reference to the Fund, and, although not directly appealing for contributions, brought home to their congregations the fact that Hospital Sunday was being honoured in London and its suburbs. Such reference would not in the slightest degree interfere with local collections or contributions: it would, however, prove a stimulus to the week-ender who is willing and able to give his share towards the Sunday Fund collections, but who through ignorance and absence is debarred from directly contributing. The London hospitals, for whose benefit the Metropolitan Fund exists, do not concern themselves solely with patients from the metropolitan area: their activities extend over a very vast field, and many a country patient has blessed them for the good that they have done. It is the least that the country can do in return to make the fact known that such and such a day is collecting day on behalf of the Fund that serves these great national institutions.

### THE DRUG MARKET.

Makers of bromides have instructed their agents not to accept orders, and this move may possibly be preliminary to a considerable advance in the prices of potassium, sodium, and ammonium bromides, but at the time of writing no announcement had been made by the makers as to prices at which they would sell. It is quite possible that the advance when it is announced may only be small; but, on the other hand, it may be substantial; in any case, those who have a fair stock on hand are to be congratulated. Apart from the interest aroused in bromide salts, the drug market has been dull and uninteresting, and few changes of any importance have taken place. The price of Epsom salts has been slightly advanced, but the increase is quite unimportant. Castor oil is again slightly cheaper. Definite and reliable reports regarding the new opium crop are awaited with interest, and business in the meantime is at a standstill.

